# Correlation between Defects and Electrical Performances of Ion-Irradiated 4H-SiC p–n Junctions

**DOI:** 10.3390/ma14081966

**Published:** 2021-04-14

**Authors:** Domenico Pellegrino, Lucia Calcagno, Massimo Zimbone, Salvatore Di Franco, Antonella Sciuto

**Affiliations:** 1 Department of Physics and Astronomy, University of Catania, 95123 Catania, Italy; domenico.pellegrino@ct.infn.it; 2 CNR-IMM, VIII Strada n°5, 95121 Catania, Italy; massimo.zimbone@ct.infn.it (M.Z.); salvatore.difranco@imm.cnr.it (S.D.F.); antonella.sciuto@imm.cnr.it (A.S.)

**Keywords:** silicon carbide, p–n junction diode, ion irradiation, point defects, defect clustering

## Abstract

In this study, 4H-SiC p–n junctions were irradiated with 700 keV He^+^ ions in the fluence range 1.0 × 10^12^ to 1.0 × 10^15^ ions/cm^2^. The effects of irradiation were investigated by current–voltage (I–V) and capacitance–voltage (C–V) measurements, while deep-level transient spectroscopy (DLTS) was used to study the traps introduced by irradiation defects. Modifications of the device’s electrical performances were observed after irradiation, and two fluence regimes were identified. In the low fluence range (≤10^13^ ions/cm^2^), I–V characteristics evidenced an increase in series resistance, which can be associated with the decrease in the dopant concentration, as also denoted by C–V measurements. In addition, the pre-exponential parameter of junction generation current increased with fluence due to the increase in point defect concentration. The main produced defect states were the Z_1/2_, RD_1/2_, and EH_6/7_ centers, whose concentrations increased with fluence. At high fluence (>10^13^ ions/cm^2^), I–V curves showed a strong decrease in the generation current, while DLTS evidenced a rearrangement of defects. The detailed electrical characterization of the p–n junction performed at different temperatures highlights the existence of conduction paths with peculiar electrical properties introduced by high fluence irradiation. The results suggest the formation of localized highly resistive regions (realized by agglomeration of point defects) in parallel with the main junction.

## 1. Introduction

Silicon carbide (SiC) has received much attention in recent years due to its large bandgap, high thermal conductivity, high breakdown field, and electron mobility [1]. All these properties make SiC an ideal material for high-power and high-frequency devices and, more generally, for the fabrication of harsh-environment electronics [2,3]. Other properties such as biocompatibility, visible blindness, and radiation hardness make this material attractive for alternative applications, such as biomedical sensors, as well as for UV and X-ray functions, charged particle detectors [4,5,6,7], and also for uses involving visible radiation presences, such as plasma environments [8,9]. 

In particular, p–n junctions, together with Schottky diodes, could certainly be considered the fundamental elements of microelectronics and as well-known solid-state devices. They have wide applications, including operations in hostile environments such as in space, and high-energy physics experiments involving charged particles and photons [10,11,12]. It is crucial to recognize the effects of particle and high-energy photon irradiation on their performances. Several reports are available that discuss the formation of irradiation defects formation in SiC [13,14,15,16,17,18,19], and it is well understood that the presence of defects implies the formation of energy states in the semiconductor band gap and could influence the electrical material properties, and consequently, the device performance. The effects of gamma, neutron, and ion irradiation on SiC devices such as Schottky diodes [20,21,22,23,24], MOS (Metal Oxide Semiconductor) capacitors [25], and BJTs (Bipolar Junction Transistors) [26] are discussed in the literature. In BJTs, point defects induced by high energy ion irradiation, already at low fluencies, are responsible for the reduction in collector current [27]. High-energy proton and electron irradiation of MOSFETs (Metal Oxide Semiconductor Field Effect Transistors) degrade the electrical performance of the device [28,29]. Some other studies in the literature report on changes in the charge collection efficiency and in the energy resolution of Schottky and/or p–n-based particle detectors induced by gamma ray, neutron, proton, and high-energy ion irradiation [7,30,31]. Other papers report on the effects of irradiation on the UV photo-response of SiC Schottky photodiodes [32,33]. Recently, we have shown [34] that defects introduced by He irradiation in a 4H-SiC-based p–n junction can be exploited to fabricate photon sources operating in the near-infrared (NIR) region.

On the other hand, the effects of ion irradiation on electrical properties of Schottky diodes and their correlation with the formation of defects have been widely investigated [13,14,15,16,17,18,19,20,21,22,23,24], although to the best of our knowledge, there are not so extensive studies in the literature exploring a wide fluence irradiation range of p–n junction diodes [35,36,37,38,39,40].

It is worth remembering that the use of irradiation defects controls carrier lifetime. Electron and proton irradiation is widely used in silicon power devices to reduce the turn-off time, the reverse recovery charge, or the recovery losses. Similarly, in SiC neutrons, electron or high-energy light ions, as well as protons or alpha particles, can be used, but their optimal use requires a full knowledge of defect structure, of its introduction rate, and of its impact on electrical device parameters in a wide range of irradiation energy and fluence [41,42].

In the present paper, we focused our study on the effects of high-energy He^+^ ions on the p–n junction by studying their electrical characteristics as a function of irradiation fluence. The aim of the paper was to investigate the effect of defect concentration and defect structure on the macroscopic properties of the p–n junction. With respect to work in the literature on BJT or MOSFET devices, our study on simple p–n junctions allowed us to examine and to understand the physical phenomena involved in a basic device where the different contributions are more easily identifiable with respect to more complex devices. The change in the macroscopic properties of the p–n junction is evidenced by current–voltage and capacitance–voltage measurements. Temperature dependence of the electrical characteristics enabled correlating the physical junction properties with changes in the semiconductor properties and the irradiation-induced defects to be determined. 

## 2. Materials and Methods

**Figure 1:**
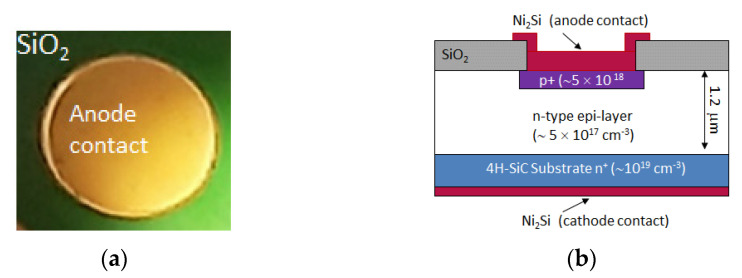
Top view picture of one diode: the circular anode contact is observable surrounded by a silicon dioxide layer (**a**); cross section scheme of fabricated devices (not to scale) (**b**).

Circular p–n diodes with diameters of 1 mm (area = 0.8 × 10^−2^ cm^2^) were fabricated in the CNR-IMM Catania clean-room facilities using a 1.2 µm thick n-type (5 × 10^17^ donor/cm^3^) 4H-SiC epitaxial layer (grown by LPE-SiC Epi Tech. Company, Milan, Italy) on an n-type heavily doped (1 × 10^19^ donor/cm^3^) 4H-SiC substrate. A top view picture and a cross section scheme of the fabricated devices (not to scale) are reported in Figure 1: the circular geometry of the device and the anode contact are observable in Figure 1a; the diode is laterally surrounded by a silicon dioxide layer, as in the scheme of Figure 1b. 

A high-dose and low-energy Al^+^ multiple implantation process was used to form a p shallow layer in the n-type epitaxial layer. A dedicated high-temperature thermal annealing process at 1700 °C for 30 min in an Ar ambient atmosphere was used to activate the dopant and to recover the implantation damage. This annealing was carried out by using a graphitic capping layer to avoid surface step bunching [4,12,43].

Previous works [44,45] have shown that the p region, obtained by the implantation process described above, has a depth of about 175 nm and an activated dopant concentration of about 5 × 10^18^ cm^−3^. Further details concerning the fabrication of the device are reported in Frazzetto et al. [44]. The ohmic contacts on the n^+^ back substrate and on the p top surface were formed by sputtering 200 nm and 100 nm thick Ni films, followed by rapid annealing processes at 1000 °C and at 900 °C, respectively, under N_2_ ambient conditions to form Ni_2_Si films [46]. 

The fabricated diodes were successively irradiated at room temperature with 700 keV He^+^ ions at different fluences in the range of 1.0 × 10^12^ to 1.0 × 10^15^ ions/cm^2^. The irradiation energy was fixed so that ions across the 1.2 µm thick epilayer and energy loss and vacancy profile concentration were almost uniform in the active device layer: the linear energy loss in elastic collision is about 2 × 10^7^ eV/ion × cm, and the linear vacancy concentration is about 10^5^ vacancies/ion × cm, as calculated by the Stopping and Range of Ions in Matter (SRIM) simulation [47].

The I–V and C–V characteristics of un-irradiated and irradiated diodes were measured in the range 300–450 K in a temperature-controlled probe station. I–V values were measured using a Source Meter Unit Keithley 2636; C–V measurements in the voltage range 0–85 V were obtained by a Boonton 7200 operating at a frequency f = 100 kHz. Deep-level transient spectroscopy (DLTS) measurements were carried out by means of a SULA double boxcar spectrometer with exponential correlator measurements in the temperature range 100–750 K by using rate windows in the range 2–200 s^−1^. 

## 3. Results

**Figure 2:**
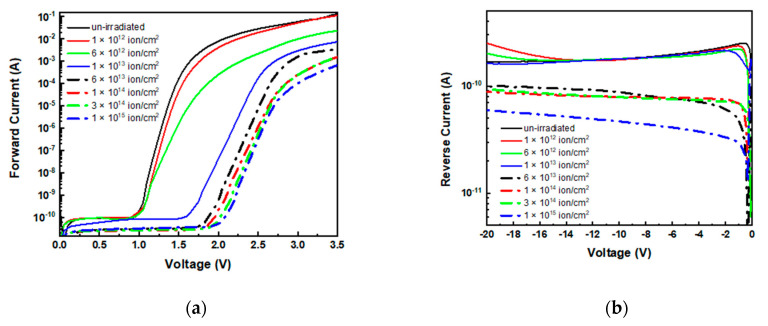
Forward (**a**) and reverse (**b**) current–voltage (I–V) characteristics of un-irradiated and 700 keV He^+^-irradiated devices at different fluencies.

The forward and reverse I–V characteristics of the p–n junction before and after irradiation at different fluences are shown in Figure 2. Relevant modifications are observable in the forward characteristics after irradiation (Figure 2a): as expected, the un-irradiated diode (black line) exhibited typical behavior with an exponential increase in current at low voltage and a linear increase at higher voltages due to the onset of the series resistance [48]. The forward characteristics changed after irradiation and we could distinguish two regions: one at low and one at high voltage. In the former, the I–Vs were governed by the recombination current, while in the latter, the series resistance of the diode was the more important parameter, as will be discussed later on.

Regarding the reverse characteristics (Figure 2b), we limited the reverse bias of investigation to −20 V, which was considered sufficient to monitor the effects of damage in the junction region, avoiding the advent of relevant lateral leakage contribute that will take place at higher polarization being absent of any guard ring. In the explored range, the current density was about 22 nA/cm^2^ in the un-irradiated diode and exhibited no relevant changes after irradiation in the fluence range 10^12^ to 10^13^ ion/cm^2^, while at higher fluences, it decreased by 3.7-fold.

The rectification ratio calculated at a bias of ±3.5 V was 5 × 10^8^ in the un-irradiated and in the low fluence-irradiated diodes, while it decreased to about 2 × 10^7^ in the higher fluence diodes, evidencing the significant degradation of diode rectifying properties.

The forward I–V characteristics of un-irradiated p–n junctions exhibited a dispersion around the typical trend (which may be due to the presence of some processing defects); however, to overcome this trouble, each device was characterized before and after irradiation and its characteristics were compared, in order to discriminate the effects due to irradiation from the processing-induced issues.

### 3.1. Series Resistance

**Figure 3:**
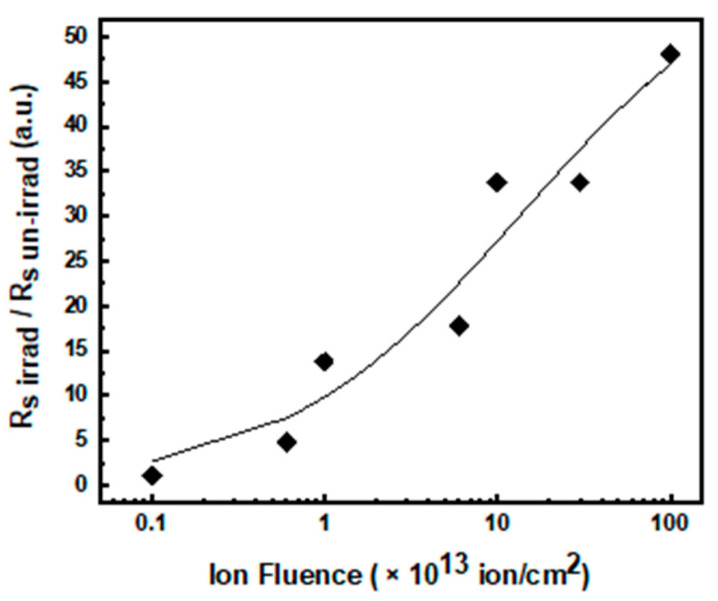
Normalized series resistance as a function of irradiation fluence.

The forward I–V characteristics in Figure 2a show the diode series resistance effects at high voltages: the curves tend to become flat, and the currents are entirely dominated by series resistances. This resistance can be considered as the sum of the contact contributions and of the internal diode resistance (related to the epilayer resistivity) [48]. All the curves show similar trend, however current inflection appears at higher bias in the irradiated diodes than the un-irradiated diode, and the current (at fixed bias) decreases by increasing the irradiation fluence. This effect can be ascribed to the rise of diode series resistance (R_s_) induced by irradiation damage. The overall diode R_s_ can be extracted from the slope of forward I–V curves in the high voltage region. We calculated the R_s_ in each device before (R_sunirr_) and after irradiation (R_sirr_), and the ratio R_sirr_/R_sunirr_ is reported in Figure 3 as a function of ion irradiation fluence: R_sirr_/R_sunirr_ exhibited an increase with the irradiation fluence. Note that the resistance increased about three-fold from the un-irradiated to the 6 × 10^12^ ions/cm^2^ irradiated device, and about 50-fold from the un-irradiated to the irradiated diode at 10^15^ ions/cm^2^.

The series resistance increase was observed in p–n junctions [36,37] as well as in Schottky diodes [21] irradiated with various types of ions and neutrons. It is associated with the increase in epitaxial layer resistivity due to the decrease in free carrier concentration [49,50]. Indeed, ion irradiation defects exhibit acceptor-like behavior [17,49], and compensation effects take place in ion-irradiated n-type SiC epilayers, inducing both decreases in carrier concentration and an increase in series resistance. Nevertheless, it is worth noting that even the decrease in carrier mobility could be responsible for the increase in R_s_. 

In our devices, the decrease in carrier concentration after irradiation was supported by the trend of the current in reverse polarization reported in Figure 2b. Reverse current was very low in the un-irradiated diodes (about 2 × 10^−10^ A @ 10 V reverse voltage) and decreased four-fold after irradiation. 

**Figure 4:**
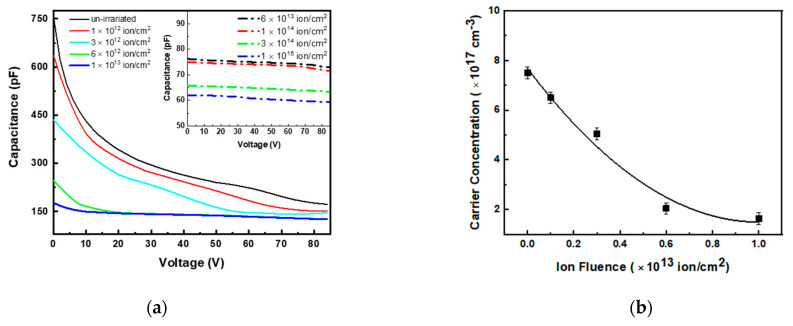
Capacitance–voltage (C–V) curves of un-irradiated and of irradiated diodes at different fluences (**a**). Dopant concentration as a function of ion fluence calculated by C–V measurements (**b**).

In order to discriminate whether the increase in the R_s_ was due to the free carrier concentration or the carrier mobility decrease, we performed C–V measurements. The C–V curves of un-irradiated and different fluencies irradiated diodes are reported in Figure 4a in the bias range 0–85 V. The reverse measurements voltage range was limited by the increase in edge leakage current due to the absence of a guard ring structure in our diodes (not included to reduce the fabrication steps, as discussed previously). 

Typical trends of C–V are observed in the un-irradiated and irradiated diodes up to a fluence of 1.0 × 10^13^ ions/cm^2^, while at higher fluence the capacitance ceased, depending on the applied voltage (insert of Figure 4a), probably due to the advent of high values of series resistance [51]. 

The carrier concentrations (N_D_–N_A_), in the n junction side, versus irradiation fluence extracted from the C–V measurements, are reported in Figure 4b: it decreased almost linearly from the initial value of 7.5 × 10^17^ cm^−3^ (which is very close to the nominal epilayer dopant concentration value) to 2.0 × 10^17^ cm^−3^ up to the fluence of 6.0 × 10^12^ ions/cm^2^. The decrease in carrier concentration by ~3.7-fold is coherent with the increase by the same factor of the series resistance (see Figure 4), indicating that carrier mobility is almost unchanged up to this value of irradiation fluence. 

At fluence ≥1.0 × 10^13^ ions/cm^2^, the C–V curves are almost constant; this behavior makes the determination of carrier concentration inaccurate (or even impossible). A similar effect was already reported by A. A. Lebedev [51] for p–n junctions irradiated with protons, and it was associated with the increase in diode R_s_. 

At higher fluence irradiation, the dopant concentration could almost certainly continue to decrease, and simultaneously, the carrier mobility could significantly reduce.

### 3.2. Recombination Current 

The forward I–V characteristics in the low voltage region can be described with the Shockley–Read–Hall (SRH) generation current and with the model reported by C.T. Sah et al. [52]. Briefly, the current is due to the recombination in the space–charge region through a deep level (deep levels) near the middle of the bandgap. In that region, the current density can be described by the relationship:

J = J_0_ exp(qV/nK_B_T) (1)

where V is the applied bias, n is the ideality factor, K_B_ is the Boltzmann constant, q is the electron charge, and T is the temperature. The pre-exponential factor J_0_ is related to the effective width of recombination region w, to the concentration of intrinsic charge carriers n_i_, and the carrier lifetime τ_0_ by the relation:

J_0_ = q n_i_w/τ_0_ (2)

It is evident from Equation (1) that any shift of I–V curves (changes of turn-on voltage) is correlated to the pre-exponential J_0_ factor, while the slope is correlated to the ideality factor n.

The J_0_ parameter is described by Equation (2), and it is related to the point defects concentration N_t_, the scattering cross-section σ, and the charge carrier speed v, with the carrier lifetime τ_0_ given by the known relation:

(3) $$\tau_{0}=\frac{1}{N_{t}\sigma \;v}$$

The obtained values of n, calculated by Equation (1) in the bias range where exponential trends occur, are reported in Table 1. This parameter is about 1.3 in the un-irradiated and 10^12^ ions/cm^2^ irradiated diodes; at higher fluences, it reaches the value n ≈ 2.1 and it is almost independent of irradiation fluence. This trend indicates the significance of the generation and recombination of charges in the space–charge region [52,53]. 

**Figure 5:**
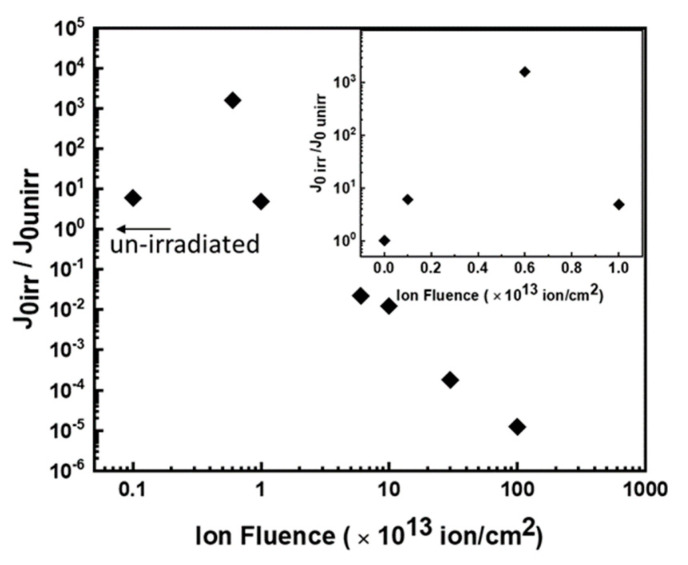
Pre-exponential factor J_0_ of irradiated devices normalized to the value before irradiation as a function of ion fluence, as obtained from the fit of I–V characteristics. In the inset, the values at low fluences are shown in detail.

We extracted the pre-exponential J_0_ values from the I–V curves of each device before (J_ounirr_) and after irradiation (J_oirr_), and the ratio J_oirr_/J_ounirr_ is reported in Figure 5 versus the irradiation fluence. This ratio increased by more than three orders of magnitude up to 6 × 10^12^ ions/cm^2^ fluence (insert), and then abruptly decreased by more than eight orders of magnitude at higher fluences. As evident from Equation (2), J_0_ depends on τ_0_, w, and n_i_, and because change in n_i_ is improbable, the modifications of J_0_ are related to the changes in w and τ_0_. 

At low fluences, the increase in J_0_ is associated both with the increase in w and the decrease in the carrier lifetime (Equation (2)). Moreover, the change in w, as reported in the literature [36], can be extracted from the barrier capacitance C_0_ (capacitance at V = 0) of the p–n junction. The effective width of recombination region w is inversely proportional to zero capacitance C_0_. This parameter after irradiation at 6 × 10^12^ ions/cm^2^ decreases by about a factor of three (see Figure 4a); therefore, we can assume an increase in w of the same factor. This suggests that the observed increase in J_0_ of three orders of magnitude is mainly due to the decreases in carrier lifetime τ_0_, i.e., caused by the increase in defect concentration, as confirmed by DLTS measurements. A decrease in carrier lifetime of about a factor of 500 is estimated.

At high fluence (≥ 1.0 × 10^13^ ions/cm^2^), we measured a decrease in J_0_ of about eight orders of magnitude, and because a decrease in point defect concentration was impossible, it must be related to the existence of further events influencing the current flow in the diode, as will be discussed later.

**Figure 6:**
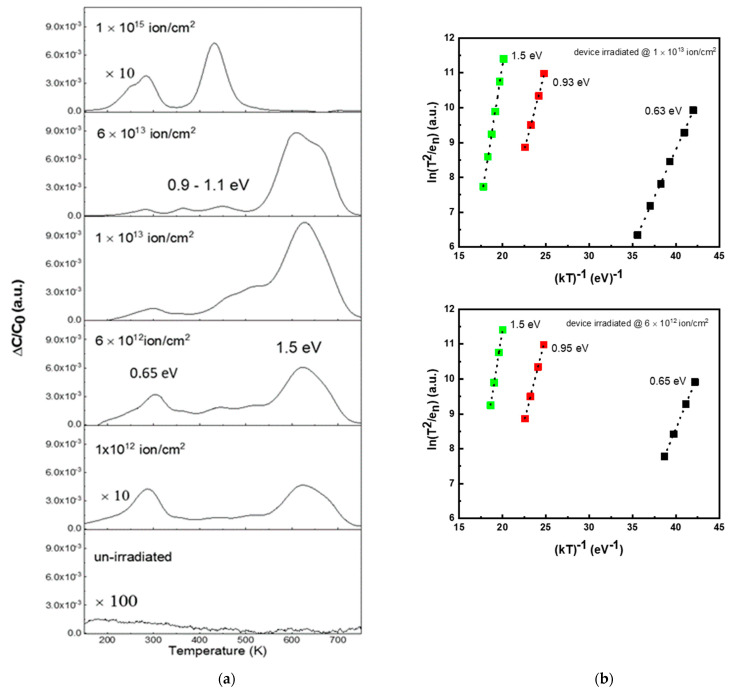
Deep-level transient spectroscopy (DLTS) spectra of un-irradiated and different fluences of irradiated devices. The energies of the main produced levels are indicated (**a**). Arrhenius plots obtained from DLTS spectra for the diodes irradiated at fluences of 6 × 10^12^ ions/cm^2^ and 1 × 10^13^ ions/cm^2^ (**b**).

The changes in the pre-exponential parameter J_0_ and the carrier concentration (and thus the series resistance studied above) are both related to the presence of deep levels associated with defects generated by irradiation. DLTS measurements were performed on all diodes in order to identify these defects. The DLTS spectra (acquired at a rate window of 23 s^−1^) are displayed in Figure 6a.

The un-irradiated diode did not show any relevant presence of traps, evidencing the good crystalline quality of the adopted epitaxial layer and the low concentration of processing defects in the n side. After irradiation at 1.0 × 10^12^ ions/cm^2^, two main peaks at 300 K and 620 K appeared in the DLTS spectra. Their energies are 0.65 ± 0.05 eV and 1.5 ± 0.05 eV below the conduction band, respectively. These energies were determined, as usual, by an Arrhenius plot of measurements performed at different rate windows. The Arrhenius plots of DLTS for 6 × 10^12^ ions/cm^2^ and 1 × 10^13^ ions/cm^2^ irradiated devices are shown in Figure 6b. The levels at 0.65 eV and 1.5 eV, reported in the literature both in as-grown and in irradiated materials, were identified as Z_1_/Z_2_ (0.65 eV) and EH_6/7_ (1.5 eV) [15,17]. Regarding the microstructure of these defects, many interpretations have been proposed: it has been stated that the Z_1_/Z_2_ and EH_6/7_ centers both originate from the same structural defect but are characterized by different charge states. Theoretical literature studies [54] and others conducted comparing theoretical models and experimental DLTS and Electron Paramagnetic Resonance (EPR) measurements [55] identified this defect as a carbon vacancy. On the other hand, some authors associated the Z_1_/Z_2_ center to a C-interstitial (interstitial carbon) or a silicon-vacancy (V_Si_), or to an antisite pair. The EH_6/7_ was attributed to a Si-related defect such as silicon–carbon vacancies (V_C_ + V_Si_), or a carbon vacancy, or a defect containing more simple defects [24,55].

A fluence of 6.0 × 10^12^ ions/cm^2^ for the intensity of the DLTS signal related to these levels increased and a further band appeared around 500 K. The literature imputes this third peak to a group of levels denominated as RD_1/2_ (“radiation damage”) with energies in the range of 0.9–1.0 eV introduced in SiC by ion irradiation [15,23]. For the RD_1/2_ centers, the exact correlation with a specific structural defect has not been established, but a V_Si_ or an antisite could be responsible for these centers [17,24]. 

The intensity of these three main levels Z_1_/Z_2_, EH_6/7_, and RD_1/2_ observed in the DLTS spectra increased with the irradiation fluence up to 6.0 × 10^12^ ions/cm^2^. The further increase in the fluence at 1.0 × 10^13^ ions/cm^2^ induced an increase in the peak intensity related to the EH_6/7_ and the RD_1/2_ levels, while the intensity of Z_1_/Z_2_ related peaks slightly decreased. The concentration of the defects for the irradiation fluence of 1.0 × 10^13^ ions/cm^2^ was in the range of 1–4 × 10^15^ defects/cm^3^. 

At higher fluence (>1.0 × 10^13^ ions/cm^2^), the EH_6/7_-related peak evolved and then disappeared, while the intensity of the other peaks decreased. This trend indicates that a rearrangement of defects occurs over the fluence threshold of 1.0 × 10^13^ ions/cm^2^. It must be outlined that at fluence greater than this threshold value, the concentration of defects could be higher than the dopant concentration and, therefore, quantitative evaluation of ion irradiation defects is not possible by DLTS. 

However, the DLTS results helped us to identify two irradiation fluence regions: (i) a low fluence regime (fluence ≤1.0 × 10^13^ ions/cm^2^) in which ion irradiation produces point defects whose concentration increases with the fluence; and (ii) a high fluence regime (fluence >1.0 × 10^13^ ions/cm^2^), characterized by a decrease in point defects concentration and, at the same time, by an interaction between primary defects. This interaction gives rise to the clustering of point defects and formation of a class of more complex defects (vacancy clusters, cluster of atoms, impurity–defect complexes, etc.). These defects are not observable by DLTS analyses, probably because they are not electrically active or because the associated band gap levels are too deep in energy to be detected with our system, as reported in the literature for Si-related defects in SiC [16].

The concentration of vacancies generated by He irradiation was estimated through SRIM simulation [47]. For the 10^13^ ions/cm^2^ fluence (experimental threshold between the low and high fluence regimes), a vacancy concentration of about 1.0 × 10^18^ cm^−3^ was obtained, corresponding to a mean distance between vacancies of about 10 nm, enough to produce defect clustering phenomena. Increasing the fluence, the defect concentration increases and the mean vacancy distance reduces: in this regime, point defect aggregation can occur. 

The discussed evolution of defects and their relative DLTS spectra are in agreement with the results on the trend of the J_0_ parameter versus the irradiation fluence resumed in Figure 4: at fluence <1.0 × 10^13^ ions/cm^2^, the increase in J_0_ with fluence (of three orders of magnitude) is mainly imputed to the accumulation of point defects. 

At higher fluence, J_0_ has the opposite behavior and decreases in many orders of magnitude, evidencing the existence of a new process influencing the current flow in the diode, ascribable to the formation of different kinds of defects. Advanced structural characterizations were performed by transmission electron microscopy (TEM) on the highest fluence-irradiated device, but they did not evidence the presence of observable defects, confirming that only point defects or very small agglomerates are produced in our device. 

### 3.3. Temperature Behavior 

**Figure 7:**
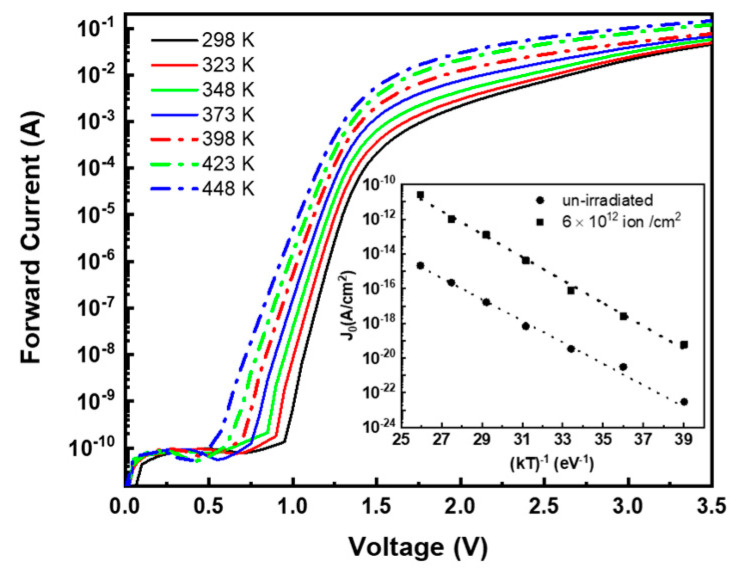
I–V characteristics at different temperatures of the 6.0 × 10^12^ cm^−2^ irradiated diode. Inset: pre-exponential factor (J_0_) obtained by the fit of I–V characteristics as a function of 1/kT for the same diode (full squares) and for the un-irradiated diode (full circles).

In order to obtain more insights into the evolution of defects in the SiC due to irradiation, I–V diode characteristics versus temperature were examined. The un-irradiated and the low fluence-irradiated devices both exhibited similar characteristics, and for brevity, in Figure 7 we report the forward characteristics of a diode irradiated at 6 × 10^12^ ions/cm^2^ measured at temperatures ranging from 298 K to 448 K. As expected, we observed a turn-on voltage decrease and an increase in forward current (at fixed bias) with the measurement temperature.

The ideality factor remains almost constant with temperature, while a change of pre-exponential parameter J_0_ is noticeable. The values of J_0_, extracted from the fit of I–V characteristics at different temperatures with Equation (1), are reported in the inset of Figure 7 versus 1/kT. It exhibits the usual exponential dependence with temperature expressed by the relation:

(4) $$J_{0}=J_{0}^{*}\;\exp\left(-\frac{E_{a}}{\mathrm{KT}}\right)$$

where E_a_ is the activation energy. From the data reported in the inset of Figure 7 (full squares), we determined the value E_a_ = 1.5 ± 0.05 eV (~Eg/2), which is consistent with the SRH model of recombination, i.e., with deep levels near the middle of the 4H-SiC energy bandgap (~3.2 eV). Similarly, for the un-irradiated diode, from J_0_ values reported in Figure 7 inset (full circles), an activation energy E_a_ = 1.43 ± 0.05 eV is obtained. 

**Figure 8:**
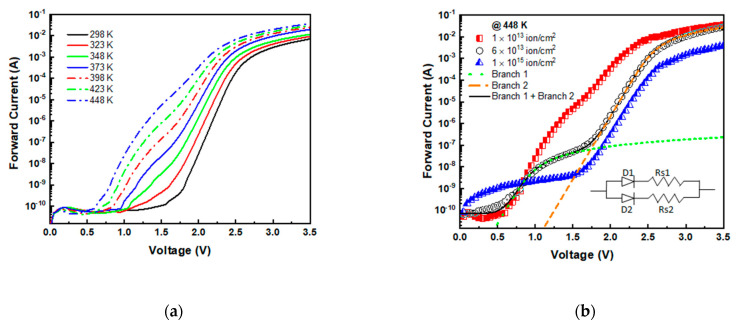
I–V characteristics at different temperatures for the 1.0 × 10^13^ cm^−2^ irradiated diode (**a**). I–V characteristic at 420 K for different fluence-irradiated diodes. The fit of the I–V characteristic with the parallel double diodes is reported for the 6.0 × 10^13^ cm^−2^ irradiated device. In the inset, a typical equivalent circuit of the studied p–n structure is sketched (**b**).

The devices irradiated at higher fluence exhibited quite different behavior, as evidenced in Figure 8a where the I–V characteristics of the 1.0 × 10^13^ ions/cm^2^ irradiated device (measured at different temperatures) are plotted. At temperatures ≥ 348 K, in the bias range between 0.5 and 1.5 V, we observed an extra current with respect to the un-irradiated device, whose value increased with increasing the temperature. Similar effects were observed in all the devices irradiated at fluence ≥1.0 × 10^13^ ions/cm^2^, and Figure 8b shows the I–V characteristics measured at 448 K for the diodes irradiated at three different fluences.

As the fluence increased, we observed a reduction in the voltage at which the extra current was triggered. These measurements also confirm the increase in series resistance already observed in the previous sections (see Figure 3). 

The presence of extra currents is reported in the literature [56,57] for as-fabricated leaky SiC p–n diodes and devices irradiated with electrons and protons (8.0 MeV). Extra currents are ascribed to epilayer defects and are due to shunt paths in parallel to the main p–n junction [58]. The I–V characteristics can be analytically described using a “parallel double diodes” structure, following the scheme sketched in the inset of Figure 8b [58]. Two parallel branches are considered: the first one contains the main diode D_1_ and a series resistance R_s1_; and a second branch contains a further diode D_2_ (differing from D_1_ in terms of J_0_) and series resistance R_s2_. We adopted this structure for the analysis of I–V characteristics versus measurement temperatures of high fluence-irradiated diodes. We assumed that the current (flowing in D_1_ and D_2_) followed Equations (1) and (2), described in previous sections. The elevated measurement temperature helps to enhance the effects of defects, allowing us to extract the contribution to the device’s electrical performances. In Figure 8b, the fit carried out on the I–V characteristics of the 6×10^13^ ion/cm^2^ irradiated diode is reported, as an example. We fixed an ideality factor of n = 2 for both D_1_ and D_2_ diodes, and the J_0_ and R_s_ values were extracted from the fit of the experimental curves. 

**Figure 9:**
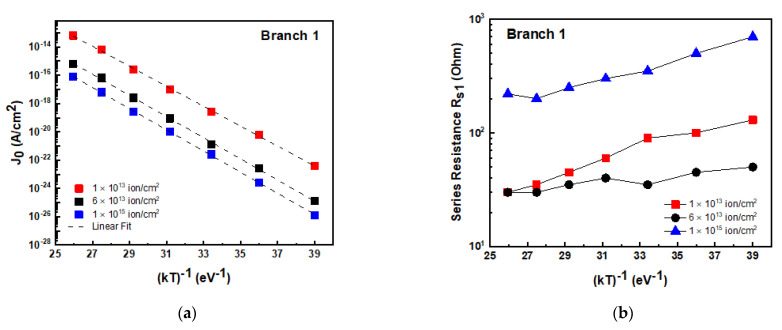
Pre-exponential factor J_0_ for diode D_1_ obtained by the fit of I–V characteristics as a function of 1/kT for high-fluence-irradiated diodes obtained by using the “parallel double diodes” structure (see text) (**a**). Series resistance R_s1_ versus 1/kT for branch 1 (**b**).

We used the “parallel double diodes” structure to fit I–V curves at different temperatures, and the J_0_ and series resistance R_s1_ values obtained for branch 1 are reported in Figure 9 versus 1/kT for the different adopted irradiation fluences. The J_0_ parameter (Figure 9a) for D_1_ diodes exhibited an exponential trend, following Equation (4), and the extracted activation energy values were in the range 1.5–1.7 eV, very close to E_gap_/2 (~1.6 eV), as expected in the SRH model. In addition, the R_s1_ (see Figure 9b) exhibited a weak dependence on the temperature for all the irradiated devices, with an activation energy of about 0.1 eV.

**Figure 10:**
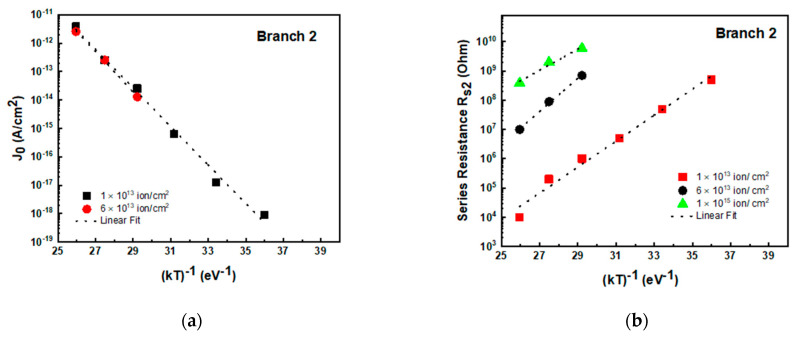
Pre-exponential factor (J_0_) for diode D_2_ obtained by the fit of I–V characteristics as a function of 1/kT for different fluence-irradiated diodes obtained by using the two-diode structure (**a**). Series resistance versus 1/kT for the same diode D_2_ (**b**).

Concerning branch 2, the J_0_ and R_s2_ extracted values are plotted in Figure 10 versus 1/kT for the same devices of Figure 9. It is worth noting that the pre-exponential factor J_0_ (Figure 10a) in D_2_ is higher than in D_1_; however, the activation energy values were E_a_ = 1.6 ±0.05 eV, very similar to that of branch 1. 

The series resistance instead exhibited a quite different trend in the second branch. As observable in Figure 10b, R_s2_ was several orders of magnitude higher than R_s1_, and it exponentially dropped by about five orders of magnitude with increasing the temperature with activation energy in the range 0.9–1.2 eV, as obtained by fitting the data of Figure 10b. It must be outlined that the determination of J_0_ (in branch 2) at high fluence is quite impossible, because the I–V curve exhibits a very low turn-on voltage (see Figure 8b).

## 4. Discussion

Ion irradiation deeply modifies the forward I–V characteristics of a p–n junction: at high voltages, the current is entirely dominated by a series resistance that increases with ion fluence due to the decrease in concentration and mobility of the free carrier. Ion irradiation defects exhibit an acceptor-like behavior, mainly inducing compensation effects in n-type SiC epilayers. 

The exponential portion of the I–V characteristics has been described by assuming that the current is governed by recombination in the space–charge region through deep level centers. An important parameter in the current description is the pre-exponential factor (J_0_), mainly related to the carrier lifetime and widely affected by the irradiation fluence. At low fluence, the carrier lifetime increases with fluence due to the growth of point defect concentrations, while at high fluence further parameters determine the current flow in the diode.

The comparison between DLTS measurements and temperature dependence of I–V characteristics indicates that the evolution of ion-irradiation defects occurs with increasing fluence. In particular, it can be argued that at fluence <1.0 × 10^13^ ions/cm^2^, simple defects are formed, whose concentration increases with the irradiation fluence; by increasing the irradiation fluence, damaged regions are generated and are responsible for conduction path formation in the active device region, and then for low-voltage extra currents observable in high-temperature I–V curves. 

In order to describe the I–V characteristics, and in particular the extra currents at low voltage, we analyzed the data by adopting a “parallel double diodes” structure. This enables analytical description of the presence of two different kinds of active defects contributing to the irradiated diodes’ forward current. In the proposed structure, diode D_1_ is related to point defects that pre-exist in the epilayer and/or are introduced by low fluence He^+^ irradiation. It has a low generation rate and low series resistance, weakly dependent on temperature. Diode D_2_ is instead related to defects produced by irradiation at fluence ≥1.0 × 10^13^ ion/cm^2^. This diode has a high carrier generation rate and high series resistance, strongly dependent on the temperature. As stated before, threshold fluence was identified as the fluence that separated the low and high irradiation regimes experimentally observed. In this last regime, irradiation induces the formation of additional defects and disorders that, on the one hand, create deep levels responsible for the generation of extra currents (through the SRH process), and on the other hand, determine the high series resistance paths advent (R_s2_ in branch 2). 

The obtained activation energy value, E_a_ = 1.6 eV ~E_g_/2 for the J_0_ parameters in both branches, confirms the SRH generation current mechanism and the correct assumption of an ideality factor n = 2. Nevertheless, the current conduction paths were quite different, as denoted by the different values and temperature dependence of series resistance R_s1_ and R_s2_ in the two branches. Experimentally, we found that R_s1_ had negligible activation energy (<0.1 eV), while R_s2_ had an activation energy between 0.9 and 1.2 eV. 

The exponential dependence of series resistance with temperature was also reported by Lebedev et al. [51] in p–n junctions irradiated with 8.0 MeV protons and was associated with a “pinning” of the Fermi level near the irradiation defects. Such behavior is also observed in other semiconductor materials, such as GaAs [59], and occurs when the defect concentration increases over the net doping concentration. In light of these considerations, we can state that the defect density in the region associated with branch 1 is lower than the epilayer doping level because the activation energy is negligible (0.1 eV). In the region associated with branch 2, however, the defect density approaches (or overcomes) the doping density and the Fermi level is pinned, leading to activation energy in the range 0.9–1.2 eV. This value is very close to the energy of the DR_1/2_ acceptor levels, probably the most important defect introduced by high-fluence ion irradiation in SiC. 

All the measurements evidenced that the conduction mechanism is the same in the two irradiation fluence regimes: at low fluence, it is essentially in the presence of point defects; at high fluence, the formation of highly resistive paths due to point defect aggregation occurs. 

## 5. Conclusions

In the present paper, the effects of He^+^ irradiation on p–n junctions fabricated on 4H-SiC epitaxial layers were examined through current–voltage, capacitance–voltage, and DLTS measurements on samples with different irradiation fluences. The electrical performances of the devices changed after irradiation and two fluence regimes were identified. At “low fluence” (≤1.0 × 10^13^ ions/cm^2^), point defects were produced by irradiation whose concentration increased with fluence and led to a decrease in free carrier concentration and lifetime. At “high fluence” (>1.0 × 10^13^ ions/cm^2^), the I–V forward curves showed a low-voltage extra current, becoming more relevant at high temperatures, and DLTS measurements evidenced a rearrangement of primary defects. The I–V characteristics were analytically discussed; they adopted a “parallel double diodes” structure consisting of a main diode with electrical characteristics, similar to the as-fabricated diode and a second diode with very high series resistance. The results suggest that high fluence irradiation produces an agglomeration of point defects which are responsible for the extra current and the formation of high-resistivity regions.

The obtained results may be applicable for an accurate control of carrier lifetime in SiC-based devices. The obtained evolution of defects can in fact be used to tune device simulations for defect engineering.

## Figures and Tables

**Table 1:** Ideality factor (n) for sample irradiated at different fluences calculated in the reported bias range.

Irradiation Fluence (Ion/cm^2^)	Ideality Factor (n)	Bias Range (Volt)
0	1.3	1.0–1.35
10^12^	1.3	1.0–1.35
6 × 10^12^	2.1	1.2–1.4
10^13^	2.2	1.75–2.3
6 × 10^13^	2.1	1.85–2.5
10^14^	2.2	1.9–2.6
3 × 10^14^	2.1	1.95–2.6
10^15^	2.1	2.0–2.6

## Data Availability

The data presented in this study are available on request from the corresponding author.
